# Objectively measured versus self-reported physical activity in children and adolescents with cancer

**DOI:** 10.1371/journal.pone.0172216

**Published:** 2017-02-16

**Authors:** Miriam Götte, Corinna Caroline Seidel, Sabine Verena Kesting, Dieter Rosenbaum, Joachim Boos

**Affiliations:** 1 Department of Pediatric Hematology and Oncology, University Children´s Hospital Münster, Münster, Germany; 2 Movement Analysis Lab, Institute for Experimental Musculoskeletal Medicine, University Hospital Münster, Münster, Germany; Universidad Europea de Madrid, SPAIN

## Abstract

**Objective:**

Existing research recognizes low levels of physical activity in pediatric patients with cancer, but much uncertainty exists about their capability to self-reflect physical activity levels. The objective of this study was to compare results of subjective self-reports and objective accelerometers regarding levels of daily walking as well as moderate-to-vigorous physical activities.

**Methods:**

Results of the objective assessment tool StepWatchTM Activity Monitor and self-reporting with a standardized questionnaire were compared in 28 children and adolescents during cancer treatment.

**Results:**

The patients were 13.8±2.8 years of age and 3.4±2.0 months after cancer diagnosis. The Bland-Altman plots indicated a fairly symmetrical under- and over-estimation for daily minutes of walking with the limits of agreement ranging from -100.8 to 87.3 min (d = -6.7 min). Mean difference for moderate-to-vigorous physical activity was almost zero but limits of agreement are ranging from -126.8 to 126.9 min. The comparison for the days with at least 60 min of moderate-to-vigorous physical activity showed a marked difference with 3.0±2.6 self-reported days versus only 0.1±0.4 measured days.

**Conclusions:**

These findings suggest that physical activity in pediatric cancer patients should preferably be assessed with objective methods. Greater efforts are needed to implement supervised exercise interventions during treatment incorporating methods to improve self-reflection of physical activity.

## Introduction

Adequate promotion of physical activities (PA) and sport is an essential prerequisite for motor development and enhances psychological and social health outcomes in children [1, 2]. Furthermore, PA and fitness during childhood are associated with numerous health benefits regarding physical (e.g. obesity) and psychological issues (e.g. depression) [3] and even with health conditions like metabolic syndrome during adulthood [4]. During cancer treatment, children and adolescents show considerably reduced levels of daily activities like walking and playing outdoors as well as significantly reduced minutes of exercise and sports per week [5]. This reduction of overall PA combined with the cancer disease and medical treatment lead to diminished motor performance and physical fitness at the end of acute cancer treatment [6, 7] and during aftercare [8, 9]. Valid tools to assess PA in this patient group are essential to define levels of inactivity and to measure the impact on activity levels in interventional studies including sports therapy. The current literature is lacking standardized methods to assess PA levels in pediatric oncology and studies either use subjective methods like questionnaires and activity scales [10, 5, 11, 12] or accelerometers [13–18]. Accelerometers are precise and valid but also complex, time-consuming and expensive and may not capture all movement whereas questionnaires and PA scales are less valid because they rely on the subject´s recollection memory and interpretation [19, 20]. However, they allow to additionally integrate more in-depth questions about subjective perceptions and values regarding exercise as wells as the precise type of exercise. Studies in healthy children and adolescents comparing subjective and objective measures of physical activity found acceptable correlations between self-reports and accelerometers for moderate physical activities but low correlations for vigorous activities with a tendency towards overestimating the active times [21, 22]. A search of the literature did not reveal any study that compared subjective and objective measures on PA in a childhood cancer population. The transparency of potential deviations between subjective self-reports and objective assessment is not only important for the design of future studies but for clinical practice because it reflects the capability of diseased children to self-estimate their levels of PA and therefore the capability to adhere to the recommendations and limits for being physically active during cancer treatment.

Aim of the present study was to compare results of subjective self-reports and objective accelerometers regarding levels of daily walking as well as moderate-to-vigorous physical activities (MVPA). We hypothesize that results of accelerometers and self-reports in children and adolescents with cancer will reveal significant deviations.

## Methods

**Fig 1:**
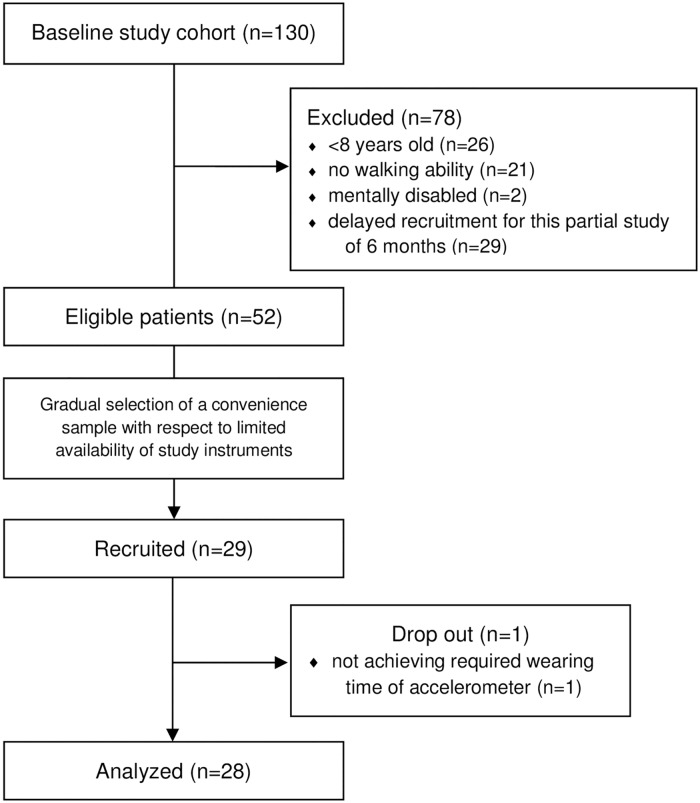
Participant recruitment.

In the course of a previous study on physical activities, a sub-group of children and adolescents (n = 29) was gradually recruited for the comparison of subjective and objective assessment of PA levels and intensities during cancer treatment (Fig 1). All patients and parents gave informed written consent for participation. The study procedures were approved by the Ethics Committee of the General Medical Council Westfalen-Lippe and the Medical Faculty of Münster (file number 2012-035-f-S). Objective measures were obtained with the Step Watch 3^™^ Activity Monitor (SAM; Orthocare Innovations, Mountlake Terrace, WA 98043, USA). This sealed uniaxial accelerometer (7.5 x 5 x 2 cm; 38 g) counts the number of gait cycles (gcs) per time interval. Gait cycles are defined as two steps and the monitor was programmed to measure in 1-minute intervals. The device is attached to the ankle with an elastic strap. The SAM is an accurate and valid tool for measuring step activity and reportedly shows an accuracy of 99,87% in children [23]. It has been validated to provide accurate measurements in persons with slow gait [24] and severe disability [25]. Applicability in pediatric patients with leukemia, lymphoma, brain tumor and bone tumor has been demonstrated [15, 18, 26]. Measurement outcomes of the SAM were the volume of activity per day (gcs per day) and intensity of activity (gcs per minute). In reference to the literature [27] 50 gcs/min (= 100 steps/min) were considered as threshold for moderate to vigorous activity levels and 20 gcs/min were defined as the threshold for continuous walking.

Subjective assessment of physical activities was conducted using the physical activity questionnaire (AQ) from the German Health Interview and Examination Survey for Children and Adolescents (KiGGS) of the Robert Koch Institute [28] with few additional cancer-specific questions. The original questionnaire has been tested for retest reliability with r = 0.83 and validity in comparison with the Sense Wear Pro2 was between r = 0.56 and r = 0.66 for leisure time sports and organized sports. The AQ consists of 45 items regarding physical activity levels before and during cancer treatment. Different questions assess daily PA (e.g. walking, playing outdoors) and moderate to vigorous physical activities (MVPA). A simple explanation of MVPA (elevated heart rate, be shortly put of breath and examples for activities) is given on the first page of the AQ. It took about 10–15 minutes to complete the questionnaire.

The comparison of SAM and AQ data was conducted for three categories: 1. Number of daily minutes of walking, 2. Number of days per week with at least 60min of MVPA and 3. Number of minutes with MVPA per week. Measurements by accelerometer (SAM) and self-report (AQ) are shown in Table 1. Participants filled in the AQ first as an unheralded self-reflection of physical activity levels without any previous motivational or educational talks. Thereafter they wore the SAM for seven consecutive days from morning after waking up until bedtime except during bathing or showering. All interested participants received an individual evaluation of their measured steps.

**Table 1:** Overview of the compared categories and respective measures of the AQ and SAM.

Category	SAM	AQ	Analysis
1: Walking (min/day)	Summarized daily minutes with > 20 gcs/min	“How much do you walk (or go with crutches) during home stays?”	Bland-Altman-Analysis, Spearman´s correlation
	*min (mean)/day*	*six answers options (from ‘never’ to ‘more than 2h/10km’ and more)*	
2: MVPA	Number of days with at least 60 minutes of >50 gcs/min	“How many days per week are you physically active for at least 60 min during home stays?”	Mean ± SD
(days with ≥60 min/week)	*days/week*	*eight answer options (0 to7 days)*	
3: MVPA (min/week)	Summarized weekly minutes with > 50 gcs/min	“Are you engaged in any sports at the moment or are you physically active in any kind? Which? How many minutes per week?”	Bland-Altman-Analysis, Spearman´s correlation
	*min/week*^a^	*type and minutes per week of up to four feasible sport activities*	

^a^ In study participants with less than seven days of wear time the hypothetical weekly score was calculated (daily mean of MVPA*7)

The accelerometer data was analyzed using the Step Watch 3^™^ Software 3.1 and raw data was exported into excel spread sheet for further analysis. Days with less than eight hours of wear time were excluded from the analysis. Commercial software (Graph Pad Prism, version 6.0) was used for statistical calculations and graphs. Bland-Altman plots were constructed to visualize the data and to assess agreement of AQ and SAM. To compare minutes of walking (category 1) of the questionnaire to the SAM data, the mean value of the marked time period (e.g. 22.5 min for marked answer 15–30 min) was used. Spearman´s analyses were computed to determine the degree of correlation between the two methods for minutes of walking and minutes of MVPA.

## Results

Descriptive characteristics of the study population are presented in Table 2.

**Table 2:** Characteristics of the study population.

Characteristics	n (%)	Mean ± SD	Range
Age (years)	28 (100)	13.8 ± 2.8	8–20
BMI (kg/m^2^)	28 (100)	20.2 ± 3.5	14.7–30.8
Gender			
Male	16 (57)		
Female	12 (43)		
Months since diagnosis		3.4 ± 2.0	1–9
Cancer type and age (years)			
Leukemia	13 (46)	12.9 ± 3.2	8–17
ALL	9		
AML	4		
Bone tumor	9 (32)	15.7 ± 2.1	13–20
Ewing sarcoma	3		
Osteosarcoma	6		
Localized at lower limb	3		
Localized at trunk/upper limb	6		
Lymphoma	2 (7)	13.0 ± 2.8	11–15
Other solid tumor	4 (14)	12.5 ± 1.3	11–14

Other solid tumor: soft tissue sarcoma (n = 2), brain tumor (n = 1), germ cell tumor (n = 1)

**Fig 2:**
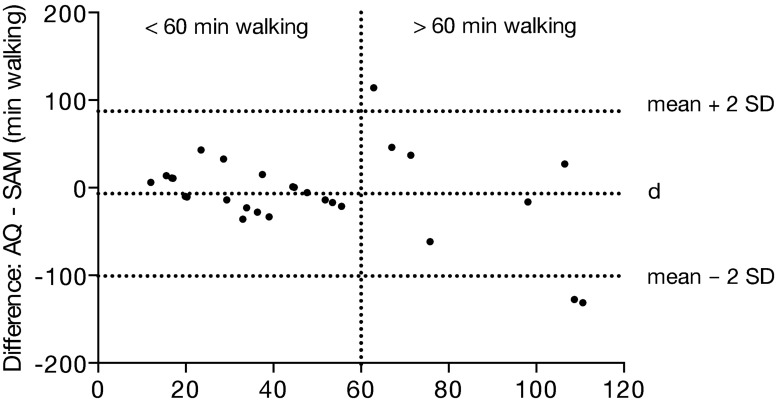
Bland-Altman plot of differences for daily walking minutes comparing AQ and SAM. x axis: mean of self-report/AQ and objective measure/SAM, y axis: differences between AQ and SAM, d = -6.7, 95% confidence limits.

All 29 included children and adolescents returned the AQ and the SAM and there were no dropouts. However, one patient did not achieve the requested eight hours of wear time on any day and was therefore excluded from the analysis. Remaining 28 participants wore the SAM for 5.8 ± 2.8 days on average. Mean wearing time was 12.1 ± 1.5 hours per day. Objective measurement with the SAM revealed a mean of 52.0 ± 44.5 minutes of walking per day and 28.6 ± 31.3 minutes of MVPA per week (4.1 ± 4.5 min per day). Total number of gcs per day was 2859 ± 2028 gcs/day. Fig 2 shows the deviations of SAM (objective measure) and AQ (subjective measure) for the category “walking” (category 1). A value below zero indicates underestimation of walking by a patient whereas values above zero indicate overestimation. The mean difference (d) is -6.7 min and the limits of agreement are ranging from -100.8 to 87.3 min indicating a fairly equal under- and over-estimation of walking minutes per day. Differences between both tools are small among those walking <60 min and large beyond the threshold of 60 min of walking. Due to the wide range and deviations in both directions (over- and underestimation) the absolute values of the differences were additionally calculated. Mean value of absolute deviations between AQ and SAM was 32.4 ± 35.5 min of walking per day. Spearman analysis indicates only a weak positive correlation between self-reported and objectively measured minutes of walking (r = 0.3; P = 0.07, graph not shown).

The comparison for the days with at least 60 min of MVPA (category 2) shows a marked difference with 3.0±2.6 self-reported days (AQ) versus only 0.1±0.4 measured days (SAM). Five patients (18%) reported to accumulate at least 60 min of MVPA on all days of the week, 11 patients reported three to six days with at least 60 min of MVPA, three patients reported one or two days and eight patient (29%) estimated that they did not meet this criterion on any day of the week. One patient forgot to answer this question. In contrast to this, the objective measurement of the SAM revealed that 26 of 28 patients (93%) did not accumulate 60 min of MVPA on any day of the week. One participant each was physically active with moderate to vigorous intensity on one and two days of the week.

**Fig 3:**
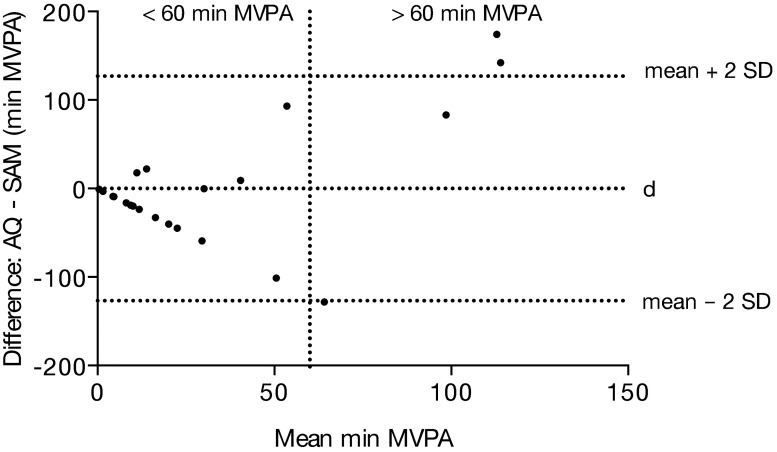
Bland-Altman plot of differences for weekly MVPA comparing AQ and SAM. x axis: mean of self-report/AQ and objective measure/SAM, y axis: differences between AQ and SAM, d = 0.05 min, 95% confidence limits.

Fig 3 compares minutes of MVPA per week as measured by SAM and AQ (category 3). The diagram shows that the mean difference (d) for MVPA measured by AQ and SAM is almost zero (0.05 min) but the limits of agreement are ranging from -126.8 to 126.9 min. Differences are small at low levels (<30–40 min) with discrepancies occurring at > 50 min MVPA. Mean value of absolute deviations between AQ and SAM was 42.1 ± 49.9 minutes of MVPA per week. Spearman analysis indicates that there is only a weak correlation between self-reported and objectively measured weekly minutes of MVPA (r = 0.1; P = 0.12, graph not shown).

Table 3 demonstrates the differences of self-estimation between patients with hematological tumors and patients with solid tumors. Correct estimation was defined as a relative deviation between self-reported and objective measure below 30% and over- and underestimation as deviations over ±30%, respectively. Almost 50% of the children with leukemia or lymphoma overestimated their daily minutes of walking whereas MVPA was more often underestimated. Patients with solid tumors, in this sample particularly bone tumor patients, tended to underestimate their levels of daily walking as well as levels of MVPA.

**Table 3:** Self-estimation of physical activity differentiated for patients with hematological vs. solid tumors.

	All patients, n (%)	Leukemia/ lymphoma patients, n (%)	Patients with solid Tumors, n (%)
**Daily walking**			
correct estimation	7 (25.0)	3 (20.0)	4 (30.8)
overestimation	10 (35.7)	7 (46.7)	3 (23.1)
underestimation	11 (39.3)	5 (33.3)	6 (46.2)
**Weekly MVPA**			
correct estimation	6 (23.1)	4 (30.8)	2 (15.4)
overestimation	6 (23.1)	3 (23.1)	3 (23.1)
underestimation	14 (53.8)	6 (46.2)	8 (61.5)

## Discussion

The present study used a standardized questionnaire as a subjective self-report and the Step Watch 3^™^ Activity Monitor as an objective tool to compare minutes of walking as well as minutes of MVPA. Our hypothesis that results from objective and subjective measurements of physical activity deviate from each other in children and adolescents undergoing cancer treatment was confirmed by the present data. Although mean differences between both tools seem to be negligible at first (6.7 min for walking and 0 min for MVPA), further analysis revealed large individual deviations that are just evened out in the mean and indicates almost equal over- and underestimation of activity levels. The results support our hypothesis that young patients with cancer have problems to reliably self-report levels of PA and deviations are increasing with amount of PA. Problems regarding self-reflection of physical activities have been reported in children with other chronic diseases like juvenile dermatomyositis and juvenile systemic lupus erythematosus [29] and the presented study reveals similar results for pediatric patients with cancer. In comparison with healthy children, the direction of self-reflection is uncommon in our study, because children typically overestimate their physical activity levels in self-reports [30, 22]. A possible explanation for poor correlations in the present study may be the changed daily routine for children during cancer treatment. Children are isolated from school and often lose contact to their social surroundings and activities. This loss of regular activity times like physical education and engagement in sport clubs presumably makes it very difficult for them to remember and categorize moments of PA. The overall low levels of PA during treatment may influence self-reports in a way that children refer their answers to their desire for being physically active. The unexpected underestimation of PA and MVPA in a relevant number of children in this study may reflect their own exaggerated feelings of being inactive due to limitations in body function and overall weakness [31]. This phenomenon seems to be especially present in patients with bone tumors and other solid tumors. More than 60% of these children underestimated their weekly minutes of MVPA by more than 30%, i.e. the accelerometer measured more minutes of MVPA than the children assumed. To support the patients´ self-confidence it might be beneficial for the children and adolescents to receive individual feedback about their daily activities by using accelerometers. This could possibly influence their own perception of abilities and physical activity in a positive way and demonstrate that even moderate to vigorous levels of PA are feasible.

Some limitations should be acknowledged regarding the study design. This study included a limited number of participants; however, with 28 children and different tumor entities a realistic insight into the topic should be possible. Furthermore, the marginal deviations (10%) regarding overall physical activity levels between the patients of this study and a comparison group from a previous study [18] indicate that the children of this study seem to be a representative cohort for pediatric cancer patients during treatment. Secondly we cannot specify the percentage of cancer treatment influencing the results in addition to the general problems in self-estimating physical activities in children. Because AQ showed good reliability and validity in previous studies with healthy children [32] it can be assumed that differences are mainly due to a loss of self-estimation capacities in children during the phase of cancer treatment. It has to be noticed that some participants have not achieved the requested seven days of SAM wear time; however according to a previous study a duration of four days of monitoring has been described as sufficient to measure habitual physical activity levels when using sealed pedometers in children [33]. To our knowledge this is the first study to assess self-reflection of physical activity levels in a cohort of pediatric patients with cancer using objective accelerometry and self-reports. There is no control group of healthy children as it was not the purpose of this study to compare self-reflection between diseased and healthy children. Therefore, the inclusion of such a control group would not have added necessary information to this study. All methods used have been tested for validity in previous studies [23–25] and for applicability in children during cancer treatment [5, 15, 18, 26].

In conclusion, objective measures should be preferentially used for the assessment of physical activities in children and adolescents with cancer to ensure accurate and reliable data. Self-reports may complementarily assess categories of activities and sports not including steps as well as expectations and attitudes towards exercise and physical activities. Findings of this study may also have important practical implications for children and adolescents with cancer. Exercise interventions during treatment should be supervised to ensure controlled and safe conditions. These interventions may also be guided with objective tools. Secondly, these interventions should incorporate methods to improve self-reflection of physical activity levels and intensity and overall body awareness, e.g. through activity monitors with biofeedback. Further research with larger samples of children and adolescents with cancer should be undertaken to investigate in detail the influences of tumor entities, age, gender and treatment phase on self-reflection of physical activity.
